# Bleeding complications of anticoagulant therapy in sepsis-induced disseminated intravascular coagulation

**DOI:** 10.1186/s13054-016-1464-5

**Published:** 2016-09-28

**Authors:** Toshiaki Iba

**Affiliations:** Department of Emergency and Disaster Medicine, Juntendo University Graduate School of Medicine, 2-1-1 Hongo, Bunkyo-ku, Tokyo, 113-8421 Japan

I thoroughly enjoyed reading the research article by Yamakawa et al. [1]. They reported the possible survival benefit of anticoagulant therapy in septic patients with coagulopathy and/or a severe condition. This result was consistent with former studies [2, 3]. However, I have some concerns regarding the increase in bleeding adverse events. Though the differences were not statistically significant, the bleeding rates tended to increase in the treated groups and the rate of bleeding requiring transfusion was between 13 and 27 %. I wonder why the incidence was so high in this study. Recombinant thrombomodulin and antithrombin were the two most popular anticoagulants in Japan and they were the dominant anticoagulants in this study. Eguchi et al. [4] reported that serious bleeding was recognized in 121 cases out of 1787 sepsis-induced disseminated intravascular coagulation (DIC) patients treated with recombinant thrombomodulin (6.8 %). We have also reported the incidence was 5.36 % in 1026 sepsis-induced DIC patients who underwent antithrombin supplementation [5]. One possible explanation for the high incidence of bleeding in Yamakawa et al.’s study was the longer observation period. Eguchi et al. and we observed the incidence of bleeding for 28 days after treatment, whereas Yamakawa et al. might have calculated it for up to 100 days (not clearly stated). Since the use of anticoagulants was usually no more than one week (thrombomodulin less than 7 days, antithrombin less than 6 days), I do not think the late phase bleeding should be included. It is reasonable to think that sustained DIC or a very severe condition lasting more than one month might contribute to an increased risk of bleeding. Since past studies revealed that inappropriate doses significantly increase the incidence, we were very careful on this point. Therefore, the recent studies performed in Japan have repeatedly demonstrated that the incidence did not increase with anticoagulant therapy [4, 5]. Thus, we would like to see the short-term bleeding incidence in Yamakawa et al.’s study. In addition, since Umemura et al. [3] reported that the incidence was substantially different depending on the agent used, information regarding which anticoagulants were responsible for the increased bleeding would also be appreciated.

## Abbreviation

DIC: Disseminated intravascular coagulation
