# Supplementary material for: Bleeding complications of anticoagulant therapy in sepsis-induced disseminated intravascular coagulation
Source: Crit Care. 2016 Sep 28;20:307. doi: 10.1186/s13054-016-1464-5 (PMC5039791; doi:10.1186/s13054-016-1464-5)
Supplement: Related texts

# Reply: Authors’ response

**Authors:** Yutaka Umemura, Kazuma Yamakawa, Mineji Hayakawa

We thank Professor Iba for his valuable and insightful comments regarding the bleeding complications associated with anticoagulant therapy for sepsis-induced disseminated intravascular coagulation (DIC). As Prof. Iba mentioned, the frequency of bleeding complications in our study [1] was relatively high compared with those in previous studies [3–5]. Prof. Iba considered that the higher frequency of bleeding complications might be attributable to the longer observation period but in our registry study incidences of bleeding were recorded only during 7 days after ICU admission. What then was responsible for the relatively high incidence of bleeding complications in our study? In the present study, we defined the bleeding complications as any bleeding events including the occurrence of intracranial hemorrhage, transfusion requirements related to bleeding, and bleeding requiring surgical intervention. However, most of the bleeding complications in this study were, indeed, transfusion requirements related to bleeding. Because transfusion therapy was applied at the discretion of the attending physician based on the treatment principles of each hospital, it is possible that different transfusion principles might lead to an unexpectedly increased incidence of transfusion requirements.

Another possible explanation for the high incidence of bleeding in our study is the combination therapy with two anticoagulant agents. Among 1247 patients in the anticoagulant group, 633 patients (50.7 %) were treated with a combination of more than two anticoagulant agents and this was associated with significantly higher risk of bleeding complications (17.2 % for combination use versus 13.0 % for single use). Also, because the risks of bleeding complications in this study were different according to the anticoagulants used (unadjusted odds ratio [OR] with 95 % confidence interval [CI] 2.65 [2.07–3.39] for antithrombin, 1.48 [1.14–1.92] for recombinant thrombomodulin, 1.98 [1.45–2.70] for serine protease inhibitors, 1.50 [0.94–2.41] for heparin), the types of anticoagulants might be responsible for the high incidence of bleeding complications in the treatment group.

Again, we thank Professor Iba for his insightful comments and providing valuable data. Bleeding complications are one of the greatest concerns associated with anticoagulant therapy. Decisions on the utilization of anticoagulant therapy depend on the balance between efficacy and safety of the therapy. We recognize that the design of the present study is not sufficient to provide definitive evidence for indications of anticoagulant therapy in sepsis. Further investigations are needed to delineate the optimal types, indications, dosages, and durations of anticoagulant therapy to gain the maximum survival benefits with minimal risk of bleeding complications.
